# Implementing Effective Infection Prevention and Control in Residential Aged Care Through Communities of Practice: Protocol for a Before-and-After Study (IMMERSE-2)

**DOI:** 10.2196/97683

**Published:** 2026-11-06

**Authors:** Joanne Tropea, Sanne Peters, Robyn Ann Smith, Lyn-li Lim, Lidia Engel, Michael J Malloy, Luke Wolfenden, Irene Blackberry, Jacqueline Francis-Coad, Wen Kwang Lim, Deirdre Fetherstonhaugh, Judy McCahon, Jacquelin McDonald, Carrie Spinks, Christopher Etherton-Beer, Chelsea Velona, Carole A Estabrooks, Noleen Bennett

**Affiliations:** 1 Department of Medicine – Royal Melbourne Hospital, The University of Melbourne, Royal Melbourne Hospital, Royal Park Campus 34-54 Poplar Road, Parkville, Victoria, 3052, Australia, 61 401338230; 2 Department of Aged Care, The Royal Melbourne Hospital, Parkville, Victoria, Australia; 3 School of Health Sciences, Faculty of Medicine, Dentistry and Health Sciences, The University of Melbourne, Parkville, Victoria, Australia; 4 Victorian Healthcare Associated Infection Surveillance System (VICNISS) Coordinating Centre, The Royal Melbourne Hospital, Parkville, Victoria, Australia; 5 Department of Infectious Diseases, Faculty of Medicine, Dentistry and Health Sciences, The University of Melbourne, Parkville, Victoria, Australia; 6 School of Public Health and Preventive Medicine, Monash University, Melbourne, Victoria, Australia; 7 School of Medicine and Public Health, University of Newcastle Australia, Newcastle, New South Wales, Australia; 8 John Richards Centre, La Trobe Rural Health School, La Trobe University, Wodonga, Victoria, Australia; 9 Western Australia for Health Ageing (WACHA), School of Health and Clinical Sciences, The University of Western Australia, Perth, Western Australia, Australia; 10 Australian Centre for Evidence Based Aged Care (ACEBAC), La Trobe University, Bundoora, Victoria, Australia; 11 Melbourne Academic Centre for Health, Faculty of Medicine, Dentistry and Health Sciences, The University of Melbourne, Parkville, Victoria, Australia; 12 Learning and Teaching Centre, University of Southern Queensland, Toowomba, Queensland, Australia; 13 Australasian College for Infection Prevention and Control, Hobart, Tasmania, Australia; 14 Western Australia for Health Ageing (WACHA), The University of Western Australia, Perth, Western Australia, Australia; 15 Translating Research in Elder Care (TREC), Faculty of Nursing, University of Alberta, Edmonton, AB, Canada; 16 National Centre for Antimicrobial Stewardship, Department of Infectious Diseases, The University of Melbourne, Parkville, Victoria, Australia

**Keywords:** infection prevention and control, community of practice, residential aged care homes, aged care nurses, practice improvement, implementation science

## Abstract

**Background:**

In Australian residential aged care, infection prevention and control (IPC) Lead nurses (IPC Leads) are a key part of the IPC program. IPC Leads are often the main IPC contacts and provide support to staff to improve IPC and resident outcomes. To enhance their competence and self-efficacy, IPC Leads need to engage in ongoing role development. Connecting with other IPC Leads, learning together, and sharing ideas and resources could also be beneficial. Communities of practice (CoPs) have been shown to improve self-efficacy and job satisfaction by fostering belonging, peer learning, and problem-solving. Drawing on social learning theory, CoPs encourage learning with and from one another to explore and iteratively develop practice. This study draws on the CoP literature and builds on our previous pilot of a CoP for IPC Leads in 1 state of Australia, which found promising impacts on confidence and practice change.

**Objective:**

This study will evaluate CoPs for IPC Leads working in Australian residential aged care. Primarily, the study will assess changes in IPC Leads’ self-efficacy. It will also explore job satisfaction and IPC practice improvement and assess the acceptability and feasibility of CoPs.

**Methods:**

We are conducting a before-and-after study using multimethods. We will recruit IPC Leads from across Australia to participate in the CoPs. The CoPs will be implemented over 12 months, with online sessions held monthly for 30 to 60 minutes each. Baseline and 12-month follow-up surveys of IPC Leads’ self-efficacy and job satisfaction and audits of IPC practice will be conducted. At the 12-month follow-up, surveys and interviews with IPC Leads will be used to assess the acceptability and feasibility of the CoPs and to explore case examples of IPC Leads facilitating practice change. Researcher notes and costing data collected will be used to further evaluate the feasibility and fidelity of the CoPs. Quantitative data will be summarized descriptively, and an ordinal mixed-effects model with a logit link will be used to assess change in self-efficacy. Interview data will be analyzed using an inductive approach, followed by a deductive approach, based on the Framework Method.

**Results:**

This project was funded in October 2024 and commenced in February 2025. As of April 30, 2026, 97 potential participants had registered their interest in participating, but no participants had been enrolled (ie, consented to participate). Recruitment of IPC Leads began in March 2026. The CoPs are expected to commence in June to July 2026. Data collection will commence in May 2026, and data analysis is expected to be completed by January 2028. Study results are expected to be published in 2028.

**Conclusions:**

The findings will create new research knowledge in the field of CoPs and knowledge translation in the aged care sector and inform the sustainability and potential for future scale-up of this collaborative social learning approach.

## Introduction

All residential aged care homes (RACHs) in Australia are required to have an effective infection prevention and control (IPC) program in place. This includes processes to appoint a nurse IPC Lead to strengthen IPC practices and outcomes [1]. Nurses in the IPC Lead role are expected to complete foundation training in IPC. The IPC Lead is the main IPC contact for the RACH. They are expected to regularly assess IPC practices, and their duties may include helping to build aged care workers’ understanding of IPC and adherence to best practice. The IPC Lead role typically seeks to increase IPC expertise across the residential aged care sector, drive IPC practice improvement, and ultimately prevent and reduce the spread of infections and improve outcomes for residents. Although the IPC Lead role is unique to Australia, IPC programs are part of the standards and regulatory requirements in aged care homes (also known as long-term care and nursing homes) internationally [2,3].

A national evaluation of the IPC Leads found that one of the key strengths of the role was the opportunity it provided for aged care nurses to upskill and gain confidence in a specialist area [4]. However, the evaluation and previous research found variation in IPC Leads’ contributions to IPC activities, including the scope of responsibilities, activities undertaken, time allocated to the role, and level of support provided, as well as limited ongoing development opportunities to enable the IPC Leads to fulfill their roles [5]. The national evaluation also found job satisfaction was suboptimal, and the retention of IPC Leads was recognized as a challenge that “...can translate to a loss in corporate knowledge of IPC.” Without opportunities for ongoing development, self-efficacy and job performance will likely be impacted. Self-efficacy, an individual’s belief in their capacity to carry out the actions needed to achieve specific outcomes, is associated with greater effort [6] and is positively correlated with nursing job performance and negatively correlated with turnover intention [7].

Communities of practice (CoPs) have the potential to improve self-efficacy and job satisfaction by fostering a sense of belonging, promoting professional development, and providing a supportive space to learn and problem-solve with peers [8]. As part of a larger study called Implementing Effective Infection Prevention and Control in Residential Aged Care (IMMERSE) [9], we evaluated a 6-month pilot of a CoP for IPC Leads in the state of Victoria, Australia. Our approach was based on the Wenger-Trayner and Wenger-Trayner [10] concept of a CoP as a social learning space to learn, think, and problem-solve with peers who share an interest in improving their practice [10]. According to this concept, CoPs have three structural elements: (1) domain (a shared interest and identity for members), (2) community (how members interact and learn together), and (3) practice (members develop a shared repertoire of resources, tools, and techniques over time).

The initial pilot CoP was co-designed and implemented in 2024 with stakeholders directly involved from the state of Victoria, Australia. The evaluation of this CoP was framed by the Value Creation Framework (VCF), a CoP-specific framework used to explore different layers of value and learning for CoP interest holders [11,12]. The pilot found that learning with and from one another was highly valued and that CoP engagement supported the IPC Leads not only to learn new knowledge but also to feel reassured in their role. Learning with and from one another helped build self-confidence, and it empowered and motivated them to share learning with colleagues and to do things differently in their workplace.

This study builds on the IMMERSE pilot. The co-design process for IMMERSE-2 (Textbox 1) builds on the foundations of the pilot project by expanding its reach nationally and updating our exploration of acceptability and feasibility in the current RACH context, which is in a period of significant change. IMMERSE-2 extends the IPC Leads CoP program across multiple Australian jurisdictions (Figure 1) and will be assessed over a 12-month period. The evaluation will build in measurement of IPC Leads’ self-efficacy, the costs of delivering the intervention (the CoPs), and audits to assess change in IPC practice. The study aims to assess changes in IPC Leads’ self-efficacy. Secondary aims are to assess job satisfaction and IPC practice change, explore examples of IPC Leads facilitating practice change, and assess the acceptability, fidelity, and feasibility of the IMMERSE-2 CoPs.

Textbox 1. **Co-designing the Implementing Effective Infection Prevention and Control in Residential Aged Care (IMMERSE-2) communities of practice (CoPs) for infection prevention and control (IPC) Leads.**

- The aims of the IMMERSE-2 co-design workshops were as follows:
  - To explore the acceptability and feasibility of CoPs for IPC Leads and other key stakeholders nationally, across all states and territories.
  - To explore possible strategies to overcome or manage any identified barriers to acceptability and feasibility.
  - To understand the current learning, development, and support needs of IPC Leads.

- Participants:
  - IPC Leads and other key stakeholders, including managers, executives, and quality staff from residential aged care homes across Australia, were invited to participate in the online workshops. Five workshops (3 with IPC Leads and 2 with other stakeholders) with 31 participants were held in October to November 2025.
- Data collection:
  - The workshops were facilitated by experienced facilitators/qualitative researchers and IPC clinician researchers from the research team (SP, JT, RAS, LLL, and NB). The facilitators asked a series of questions guided by the Theoretical Framework of Acceptability (TFA) [9]. The TFA includes 7 constructs—effectiveness, affective attitude, ethicality, burden, intervention coherence, opportunity costs, and self-efficacy. Additional questions explored the feasibility of CoPs, including their length, frequency, facilitation, scope, learning needs, and potential practice focus. Five online workshops were held, each 1 hour in duration. They were recorded and transcribed verbatim for analysis.
- Data analysis:
  - The workshop transcripts were analyzed using an inductive thematic analysis followed by a deductive coding approach. Initially, 3 researchers coded the first workshop together to formulate a coding strategy guided by the TFA. Coding of the remaining workshops was completed by 1 researcher, with uncertainties reviewed by 2 senior researchers.

- Due to a lack of consensus regarding priority IPC topics for the CoPs, additional interviews exploring IPC topics were conducted with 3 IPC experts from large national aged care providers.

- Main findings:
  - Strong support for CoPs for IPC Leads: Participants responded positively to the idea of a CoP, seeing it as a valuable way to connect, share practical solutions, and reduce the sense of working in isolation. Many felt that CoPs would help strengthen IPC knowledge, confidence, and consistency across the sector.
  - Importance of practical, relevant, and engaging content: Participants emphasized sessions should be interactive, solution focused, and tailored to the real challenges of aged care. Sharing case examples, tools, resources, and outbreak experiences was viewed as particularly helpful.
  - Time and workload are the major barriers: Across all workshops, the biggest concern was the lack of dedicated time for IPC responsibilities. Competing priorities, staffing pressures, and difficulty getting off the floor often limit participation in learning activities, including attending the CoP.
  - Equity, confidentiality, and inclusion matter: Participants highlighted the need for safe and confidential discussions, especially when sharing sensitive issues. Ensuring representation from diverse services—including culturally and linguistically diverse, regional, remote, and standalone facilities—was also viewed as important.
  - Support from leadership is essential: Attendance and engagement depend on organizational support, adequate staffing, and clear recognition of IPC Lead responsibilities within role descriptions. Many participants agreed that a letter or flyer addressed to the residential aged care home executives would help communicate the benefits of supporting IPC Leads and encourage their participation in the CoPs.
  - CoPs are expected to provide several benefits: These include building confidence, especially for newer IPC Leads; providing practical guidance missing from traditional training; encouraging consistent practice, shared problem-solving, and resource sharing; and supporting preparedness for outbreaks and emerging issues.
  - The preferred logistics include short, structured online sessions: Most participants preferred sessions supported by agendas distributed in advance, recordings, shared resources, and opportunities to ask questions and engage in general IPC discussions.
  - The main topics of interest include the following: Antimicrobial stewardship and transmissible infections beyond COVID-19.

**Figure 1.**
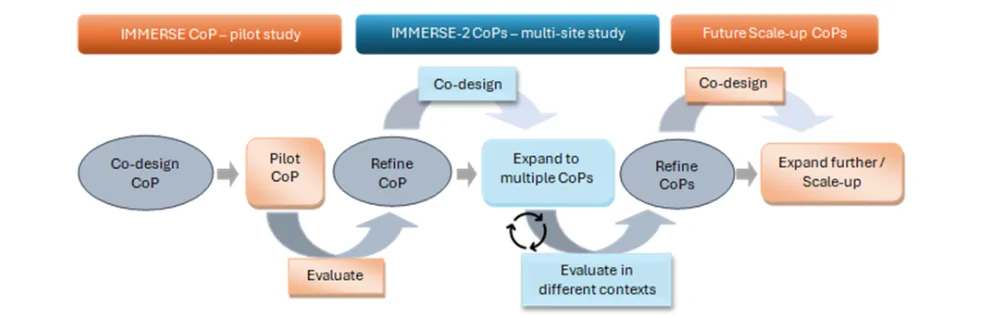
Outline of Implementing Effective Infection Prevention and Control in Residential Aged Care (IMMERSE), IMMERSE-2, and future scale-up. CoP: community of practice.

## Methods

### Study Design

We are conducting a before-and-after study using multimethods to evaluate CoPs for IPC Leads, implemented over 12 months, with a focus on the primary outcome (see Table 1 for an outline of the study schedule).

This study builds on the IMMERSE work, in which self-report and scenario-based assessments were used to measure current IPC practice, as audits were not within the scope of that project. For this study, we propose building in observational audits and chart audits among a random sample of participants to assess IPC practice before and after the IPC Leads participate in the CoPs. CoPs will be offered in 2 distinct IPC topic areas, and IPC Leads will join 1 topic group. The random sample of participants from 1 topic group will serve as the control group for the random sample of participants from the other topic group. This will provide a component of comparative design within the study.

**Table 1.** Study schedule [13].

Study activity	Study period														
	Enrollment		Postallocation												Close-out
	–t_i_ to 0	0	t_1_	t_2_	t_3_	t_4_	t_5_	t_6_	t_7_	t_8_	t_9_	t_10_	t_11_	t_12_	
Enrollment															
Eligibility screen	✓														
Informed consent	✓														
Group allocation		✓													
Intervention															
CoP^a^ sessions			✓	✓	✓	✓	✓	✓	✓	✓	✓	✓	✓	✓	
Assessments															
IPC^b^ Leads survey^c^	✓														✓
Audits			✓												✓
Session feedback survey			✓	✓	✓	✓	✓	✓	✓	✓	✓	✓	✓	✓	
Acceptability survey								✓							✓
Interviews															✓

a CoP: community of practice.

b IPC: infection prevention and control.

c The survey includes an assessment of self-efficacy, job satisfaction, and IPC practice.

### Ethical Considerations

Ethics approval for the conduct of this study was obtained from the University of Melbourne Human Research Ethics Committee (Project ID: 33094). Informed consent will be sought from IPC Leads who participate in the study, as well as RACH managers for consent to participate in the audits. Privacy and confidentiality will be maintained by storing data in a password-protected folder accessible only to the named researchers. Findings will be summarized and presented in a nonidentifiable way.

### Participants and Recruitment

The sampling pool for this study will be all RACHs in Australia (N>2600 homes and ~750 providers). Our recruitment methods will be purposive to ensure representation from different provider groups (public, private, and not for profit) and locations (metropolitan, regional, and rural locations), and all Australian states and territories. We will promote the CoPs nationally at aged care and IPC conferences, via industry and professional association websites, publications, and newsletters (eg, Aging Agenda and the Australasian College for Infection Prevention and Control), and send email invitations directly to RACH managers and executives.

The inclusion criteria are as follows: the study population will comprise IPC Leads employed in RACHs across Australia. Eligible participants must be registered nurses or enrolled nurses currently working “on the floor,” providing care to residents in a RACH, and appointed to the IPC Lead role, which includes responsibility for overseeing IPC practices within their facility. Participants must also be willing to take part in a 12-month CoP, which involves attending regular sessions and participating in evaluation activities, such as surveys and interviews. Informed consent will be obtained from all participants.

### Intervention: IMMERSE-2 CoPs for IPC Leads

The design of the IMMERSE-2 CoPs for IPC Leads has been informed by findings from our CoP pilot study, the IMMERSE-2 co-design workshops, and stakeholder interviews held in October and November 2025 (see Textbox 1 for a summary of the IMMERSE-2 co-design activities).

We plan to establish 4 or more CoPs with 100 plus IPC Leads (≥25 IPC Leads recruited per CoP) representing multiple RACHs and providers located across 4 or more Australian jurisdictions. This number of CoPs was selected to offer coverage for jurisdictions around Australia, to allow for time differences and topic options, and to remain feasible. The number of IPC Leads to be recruited is largely based on experience from the IMMERSE pilot. It accounts for the likelihood of dropouts and is also informed by the co-design workshops.

Each CoP will focus on one of the following 2 IPC topics that were identified as high priorities for IPC Leads: (1) antimicrobial stewardship (AMS) and (2) transmissible infections. Participants will be asked to select the IPC topic group they would like to join and nominate preferred days and times for the CoP to meet. The first CoP session will be used to introduce members, start planning the key aspects of the IPC topic that members would like to cover, and identify what each group hopes to achieve over the 12 months. Sessions will be held on Microsoft Teams and will be recorded to enable the research team to monitor the content and summarize each session. To encourage real-time participation, session recordings will not be available to IPC Leads. A summary of each session, with links to the resources discussed, will be provided after each session.

Initial sessions will be facilitated by a member/s of the research team. CoP members will be asked to self-nominate to take on the facilitator role, aiming for at least one facilitator per group. The facilitators will be supported by the research team. This includes meeting with a researcher prior to each session to draft a run sheet and cofacilitating sessions as needed. The level of ongoing support will be tailored to the needs of the facilitators.

### Measures

The primary outcome measure is change in IPC Leads’ self-efficacy from baseline to 12-month follow-up. Secondary measures include job satisfaction, IPC practice change, case examples of IPC Leads facilitating practice change, and implementation measures of CoP feasibility, including reach and intervention costs, acceptability, and fidelity.

Variables of interest: as part of the baseline survey, we will collect data (descriptors of the study sample) on IPC Lead characteristics and workplace, including roles/positions; years of experience working in residential aged care, at the current home, and as IPC Lead; completion of IPC training; the presence of an IPC Lead position description; the number of allocated hours for the IPC Lead role; the hours spent on IPC Lead responsibilities; the supports and resources accessed; overall knowledge of IPC; overall confidence in the role; and confidence in IPC activities (1=“poor” to 5=“excellent”). Information will be collected on the geographical location, size (number of resident beds), provider type, and whether audits of IPC practice are routinely conducted (type of audits, who conducts these, and how they are reported).

### Data Collection

Table 2 summarizes the data collection.

**Table 2.** Summary of data collection involving IPC^a^ Leads and RACH^b^ staff.

Focus	Participants	Time points	Tools/methods	Data source
Self-efficacy	IPC Leads	Baseline and 12-month follow-up	GSE^c^; modMJS^d^	Survey
Job satisfaction	IPC Leads	Baseline and 12-month follow-up	modMJS	Survey
Facilitating IPC practice change	IPC Leads	12-month follow-up	Case examples	Surveys and interview data
IPC practice	RACHs (random sample)	Baseline and 12-month follow-up	Audit of antimicrobial use; observational audit of IPC practice	Medication records; simulated practice
Reach	IPC Leads	Recruitment phase	Recruitment rates; participation rates; member demographics and characteristics	Registration forms; researcher log; session recordings
Feasibility: experience, barriers, and enablers	IPC Leads	Postsessions 12-month follow-up	Experience (satisfaction); barriers and enablers to participating in the CoPs^e^	Survey and interview data; survey
Intervention costs	—^f^	During the intervention phase and at 12-month follow-up	Cost to delivering the intervention; resources required	Researcher log; micro costing checklist
Acceptability	IPC Leads	6-month and 12-month follow-up	Experience (satisfaction), attitudes, and burden (reasons for dropout and nonparticipation)	Surveys
Fidelity	—	During the intervention phase and at 12-month follow-up	Key components of the intervention (levels of completion)	Researcher log; audit session recordings

a IPC: infection prevention and control.

b RACH: residential aged care home.

c GSE: general self-efficacy.

d modMJS: modified Measure of Job Satisfaction.

e CoP: community of practice.

f Not applicable.

#### IPC Leads’ Self-Efficacy

At baseline and 12-month follow-up, we will assess IPC Leads’ self-efficacy to fulfill their role in their workplace using the General Self-Efficacy Scale (GSE) [14]. The GSE was selected as it is a validated 10‐item instrument that has been used across multiple studies and settings. IPC Leads will be asked to rate how true each of the GSE items is for them in their IPC Lead role using a 4‐point Likert scale response from 1=“not at all true,” 2=“hardly true,” 3=“moderately true,” and 4=“exactly true.” The total score, calculated as the sum of the individual item responses, ranges from 10 to 40, with a higher score indicating better self‐efficacy levels.

#### IPC Leads’ Job Satisfaction

At baseline and 12-month follow-up, the modified Measure of Job Satisfaction (modMJS) will be used to assess IPC Leads’ job satisfaction [15]. The modMJS has been tested among residential aged care staff in Australia and consists of a 5-factor, 22-item instrument. It uses a 5-point Likert scale ranging from 1=“very dissatisfied,” 2=“dissatisfied,” 3=“neither dissatisfied nor satisfied,” 4=“satisfied,” and 5=“very satisfied.” The total score, calculated as the sum of the individual item responses, ranges from 22 to 110, with a higher score indicating greater job satisfaction. We will also document the retention and turnover of IPC Leads.

#### IPC Practice Change

Audits of antimicrobial usage and transmission-based precautions will be collected from a random sample of participants, stratified by IPC topic group, location, and provider group type. The audits will be based on national guideline recommendations [16]. These data will be collected through a retrospective audit of medication charts and real-time observational audits at baseline and repeated at the 12-month follow-up.

Specifically, it will assess the incidence of antimicrobial days of therapy (DOT) per 1000 resident days and the prevalence of PRN (pro re nata) orders for antimicrobials. Participants will be asked to provide the total number of residents per day and, for each antimicrobial prescribed, the name of the antimicrobial, the route of administration, whether it was prescribed PRN, and the number of days it was prescribed during a specified 28-day period (eg, May 1-28, 2026, inclusive). All data provided will be nonidentifiable.

Observational audits of RACH staff (n=3‐5/home) performing a mock clinical task requiring transmission-based precautions will be conducted. We will modify existing observational audit tools [17,18]. The number and percentage of correct observations will be measured. Observational audit data will be collected in-person by research staff. To ensure consistency in observational audit data collection methods, the researchers conducting the audits will receive training from IPC experts on our research team (NB, LLL, and CS).

Baseline and follow-up audits will be conducted at 10% (a minimum of 2 RACHs per CoP) of IPC Leads’ RACHs. Audit data collected from those in the AMS topic groups will act as the control group for the transmissible infections topic groups and vice-versa. Reductions in antimicrobial usage (reduced DOT and reduced PRN use) from baseline to follow-up and a greater proportion of correct observations from baseline to follow-up are considered improvements in IPC practice.

#### Facilitating Practice Change

At the 12-month follow-up, IPC Leads will be asked to self-report case example/s of initiating and facilitating IPC practice change via open-ended questions on the follow-up survey, or in more detail, via semistructured interviews (Multimedia Appendix 1). They will be asked to describe how they applied what they learned from the CoP back at their workplace and what difference (if any) this was perceived to have made. All IPC Leads who take part in the CoPs will be invited to participate in a follow-up interview.

#### Reach

Reach will be assessed by calculating the number and proportion of IPC Leads recruited to the CoPs and the number of IPC Leads who participate in the CoPs. The representativeness of individual IPC Leads will be assessed using baseline data on their demographics (gender, years’ experience, and roles) and the characteristics of the RACHs.

#### Feasibility: Experience/Satisfaction

After each CoP session, participants will be asked to rate their satisfaction with the following four items: (1) learning experience, (2) content covered, (3) opportunities to engage with others, and (4) technology; from 1=“very dissatisfied” to 5=“very satisfied.” They will be asked 2 open-ended items “Something I will take away from today...” and “Please share any comments, suggestions, questions or feedback about the CoP or IPC.” The postsession survey was developed and applied in the pilot CoP program and refined for this study.

Barriers and enablers to IPC Leads’ participation, and the feasibility of the CoPs, will be explored in the follow-up survey and interviews. Survey items and semistructured interview questions will be guided by the capability, opportunity, motivation, and behavior (COM-B) model, exploring factors associated with capability (C), opportunity (O), and motivation (M) to participate [19].

#### Intervention Costs

Data on resources and associated costs required to deliver and participate in the CoPs will be recorded in a purpose-built spreadsheet. This will include information on role (facilitator, participant, trainer, and administration), time spent (in minutes), time of work (during or after working hours), type of activity (preparation, participation, postsession follow-up), and a brief description of what was done. Cost data will also be collected from the health and aged care provider perspective and valued using standard national wage rates and other relevant cost sources. All costs will be expressed in Australian dollars for the reference year 2026.

#### Acceptability

At the 12-month follow-up, acceptability of the CoP will be assessed via surveys. Questions will be guided by the Theoretical Framework of Acceptability (TFA) and a TFA-based questionnaire [20,21]. The TFA questionnaire consists of 8 items, with each item scored on a 5-point Likert scale 1=“strongly disagree,” 2=“disagree,” 3=“neutral,” 4=“agree,” and 5=“strongly agree,” with higher scores reflecting greater acceptability. The survey will include an additional open-ended question asking participants, “How do you think that the acceptability can be enhanced?”

#### Fidelity

Key components of the intervention will be measured by levels of completion. Attendance rates per session and the frequency of CoP sessions will be monitored by the research team. Recorded CoP sessions will be audited by trained research staff to assess the level of interactions, including verbal and nonverbal communication and interactions via audio and the online chat function.

Fidelity will include whether the following are achieved: (1) 4 or more CoPs are established and operate over a 12-month period; (2) 100 or more IPC Leads are recruited to participate in the CoPs (≥25 in each CoP); (3) IPC Lead members represent 4 or more Australian jurisdictions, multiple RACHs, and providers; (4) 10 or more CoP sessions are held over 12 months for 30 to 60 minutes each; (5) at least 4 IPC Leads from different RACHs attend each CoP session; and (6) members attend at least 50% of sessions. In accordance with previous research [22], the fidelity of each element will be defined as high (80%‐100%), moderate (51%‐79%), and low (<50%). Other measures of fidelity will be developed following the first session of each CoP, during which members of the CoPs will plan and confirm how the CoPs will be delivered.

### Data Analysis

Descriptive statistics will be used to summarize participant characteristics from the baseline survey, recruitment and participation rates (reach), fidelity, and postsession survey data.

#### IPC Leads’ Self-Efficacy and Job Satisfaction

Responses from individual Likert survey items of the GSE (primary outcome) and modMJS will be assumed to come from an ordinal scale with equal spacing between categories. For each factor consisting of multiple items, a total score will be calculated by summing across all items for a given factor. To test for changes in IPC Leads’ self-efficacy and job satisfaction from pre to post CoP, an ordinal mixed-effects model with a logit link will be used. Time (overall pre-post change) and CoP will be fitted as fixed-effects, with each IPC Lead identifier fitted as a random-effect to account for any within-person correlation.

#### Facilitating Practice Change

Data on case examples of facilitating practice change from interviews and open-text survey responses will be analyzed using an inductive approach, followed by a deductive approach, based on the Framework Method [22]. This method has clear and structured steps that support collaboration between multiple researchers. The Framework Method has seven steps: (1) transcription; (2) data familiarization; (3) line-by-line inductive coding to identify emerging themes in the data; (4) development of a working analytical framework in which all inductive themes or codes will be combined with deductive codes according to the VCF [11]; (5) application of the analytical framework (inductive and deductive coding structure) to all data; (6) charting of data into the framework matrix. The matrix will structure data (coded transcripts) into columns (codes) and rows (participants). This will be done in Excel or NVivo (Lumivero) software. The charting process involves a thoughtful balance of summarizing the data without removing the original meaning; and (7) data interpretation will be iterative, code descriptions will be created and example quotes will be selected to describe participants’ experiences. The VCF explores indicators of potential value in 8 domains that include contextual factors, learning, practice, and community outcomes of the CoPs. Steps 1 to 7 will be conducted by 2 researchers in close collaboration with each other, and key methodological decisions will be recorded and discussed with the research team. Steps 4 and 7 will be critically reviewed during frequent research team meetings to discuss and revise developing themes and verify that interpretations are closely linked to the data.

#### IPC Practice Audits

To measure the incidence of total, oral, and topical antimicrobial use in audited RACHs, antimicrobial DOT per 1000 resident days will be calculated. The comparison of DOT rates at baseline and at the 12-month follow-up by CoP topic group will be analyzed using mixed-effects Poisson regression with a log link, robust variance to account for any mild misspecification, and resident days as the offset. Fixed effects fitted include the CoP topic group and time (overall pre-post change) as well as their interaction, with RACH identifiers fitted as a random intercept to account for any differences within RACHs. If overdispersion is identified, a negative binomial model will be used instead. The number of PRN antimicrobial orders will be described using frequencies and percentages of residents prescribed one or more PRN orders of an antimicrobial in the previous month.

For the observational audit of transmission-based precautions data, descriptive statistics will be generated for the number of correct steps by CoP topic group (median and IQR). To compare proportions, a mixed-effects logistic regression model will be applied. Fixed effects fitted include the CoP topic group and time (overall pre-post change), as well as their interaction, with RACH identifiers fitted as a random-intercept to account for any differences within RACHs.

#### Feasibility: Barriers and Enablers

Data on barriers and enablers from interviews and open-text survey responses will be analyzed using an inductive followed by a deductive approach based on the Framework Method, as described above. The capability, opportunity, motivation, and behavior will be used to guide the deductive analysis.

#### Intervention Costs

The cost per CoP and the total cost of the intervention will be calculated over the 12-month intervention period.

#### Acceptability

Data analysis of the TFA items on the follow-up survey will be summarized using descriptive statistics. The criterion for success is that more than 70% of participants will respond with either “agree” or “strongly agree” to the general acceptability survey questions.

#### Integration of Findings

Where multiple data sources are used to assess the study aims, findings will be merged for further analysis to contextualize meaning and interpret findings [23].

## Results

This project was funded in October 2024 and commenced in February 2025. As of April 30, 2026, 97 potential participants had registered their interest in participating, but no participants had been enrolled (ie, consented to participate). Recruitment of IPC Leads began in March 2026, and enrollment commenced on May 14, 2026. The CoPs are expected to commence in June to July 2026. Enrollments will be open until the date of the third CoP session (September 2026). Data collection will commence in May 2026, and data analysis is expected to be completed by January 2028. Study results are expected to be published in 2028.

## Discussion

This study builds on the pilot CoP for IPC Leads that we conducted in the state of Victoria, Australia. It will provide further insight into how to co-design, deliver, and evaluate CoPs for health care professionals who work in RACHs from a diverse range of providers and settings across Australia. There is limited research on how CoPs can impact practice change in residential aged care [24,25]. This will be one of the few studies to comprehensively evaluate CoPs among residential aged care workers from multiple providers, care homes, and Australian jurisdictions. Using multimethods, the evaluation will focus on a range of measures to assess change (including IPC Leads’ self-efficacy, job satisfaction, case examples of facilitating practice change, and IPC practice) and implementation measures (including acceptability, feasibility, and intervention costs).

Using co-design to develop the CoPs aims to enhance uptake and acceptability among IPC Leads [26]. Co-design workshops were held with IPC Leads and senior staff who support them in their roles, including quality staff and managers. Senior staff’s awareness and acknowledgment of the value of the CoPs may facilitate IPC Leads’ engagement in the CoPs. Feedback from IPC Leads will also be collected iteratively throughout the whole project, for example during the first introduction session and after each CoP session, to draw insights that may further enhance their ongoing uptake and participation. The CoPs will focus on 2 IPC topics—AMS and transmission-based infections. These topics were decided based on information gathered during the co-design process.

The IPC Lead role was introduced in Australia on December 1, 2020, during the height of the COVID-19 pandemic. Since that time, the role has evolved in response to recommendations from the national evaluation of IPC Leads and the strengthened Aged Care Quality Standards, which took effect on November 1, 2025 [4,27]. This includes changes to IPC Lead training and reporting and new measures for IPC systems. Recommendations from the national evaluation include regional CoPs to complement support platforms, IPC Leads undertake continuous professional development activities focused on IPC, and the integration of the IPC Lead role and activity outputs, such as audits and the communication of new IPC information, into governance structures. The IMMERSE-2 CoPs have the potential to help IPC Leads and their organizations meet these key recommendations.

The IMMERSE-2 CoP will bring IPC Leads together to connect, learn with and from one another, and problem-solve together. While the CoP will focus on IPC practice, it will also provide the opportunity for capacity building in skills such as leadership and driving practice change. These skills are not IPC specific and therefore transferable to other clinical and care areas in the sector. Additionally, CoP members who take on the facilitator role will develop skills in planning and time management, active listening and effective questioning, and summarizing and paraphrasing group discussions.

We recognize there are some limitations with the study design. The main study limitation is the lack of a control group to compare findings. Instead, we are using a before-and-after study design without a control to increase uptake and participation in the CoPs. Second, there are some limitations with the use of observational and record audits of IPC practice. The audits will be conducted in a random sample of aged care homes, as it is not feasible to collect these data from all participating RACHs. We recognize this will capture a snapshot of IPC practice, and that there is also the risk that the presence of the observer will influence practice. A strength is that the topic-specific CoPs will be each other’s control because both AMS and transmissible infection audits will be measured in the random sample.

This study provides an opportunity to transform the care provided to older people living in RACHs by supporting IPC Leads to facilitate practice improvement and by working with IPC Leads to share knowledge and experiences through a collaborative CoP model. The study findings will inform the sustainability and future scale-up of the CoP for residential aged care nurses.

## Supplementary material

10.2196/97683 Multimedia Appendix 1 Interview guide with members of the infection prevention and control Leads community of practice.

## Abbreviations

AMS: antimicrobial stewardship
CoP: community of practice
DOT: days of therapy
GSE: General Self-Efficacy Scale
IMMERSE: Implementing Effective Infection Prevention and Control in Residential Aged Care
IPC: infection prevention and control
modMJS: modified Measure of Job Satisfaction
PRN: pro re nata
RACH: residential aged care home
TFA: Theoretical Framework of Acceptability
VCF: Value Creation Framework
