# Supplementary material for: Implementing Effective Infection Prevention and Control in Residential Aged Care Through Communities of Practice: Protocol for a Before-and-After Study (IMMERSE-2)
Source: JMIR Res Protoc. 2026 Nov 6;15:e97683. doi: 10.2196/97683 (PMC13742776; doi:10.2196/97683)
Supplement: Supplementary file 1 [file resprot-v15-e97683-s001.docx]

## **Follow up interview guide with members of the IPC Leads CoP (Semi Structured interview)**

Thank you for your time today. There are three things we’d like to talk about with you today: 1) your CoP experience; 2) your feedback on how suitable/potentially useful a CoP is for residential aged care staff/setting; 3) feedback on any practice improvement activities you led or worked on.

There are no right or wrong answers, we’re interested to understand your experiences being part of the CoP, and your experiences in the IPC Lead role. If at any time you do not wish to answer a question or feel uncomfortable, please let me know and we can go to the next question or take a break.

1. We’d like to hear about your experience of the Community of Practice
   a. Tell me how the CoP experience has made you feel…..? Activities/sessions you did or didn’t enjoy; explore experience of the structure/format/content/facilitation of the CoP.
   b. Tell me about any new insights, knowledge, resources, ideas, tools or methods you’ve learned, and/or social connections you’ve made.......
   c. How do you use what you’ve learned in practice? What do you “do” differently? Is there anything you’ve changed/modified?
   d. The changes you’ve applied to make a difference to ‘what matters’ – the effect, impacts and outcomes that happen for residents, carers or other staff members...(explore any differences made and for whom. Probe for specific examples).
   e. How does this community interact and engage with (or not) this wider landscape – in your facility, between facilities etc.
   f. Tell me about the resources, people, developing as a group, what has helped make all this possible….....what has been a barrier to the CoP operating well?
   g. What engagement with stakeholders has (or could) help/ed others understand the importance of this work…..…? Who has been involved....?
   h. In what new ways (if any) are you thinking about your work or yourself?

(Explore these if not already covered above or responses to similar survey items are low or lack detail – check for any further insights/feedback)

2. What worked well about the CoP?
3. What could be improved about the CoP?
4. Has being part of the CoP helped you in your IPC Lead role? If so, how? Can you describe this in more detail? If not, what, if anything could be done differently for the CoP to be more helpful?
5. Did you interact with anyone from the CoP in between sessions? Please tell me more about this (explore why, frequency, what was sought/offered, details about the connection).
6. What are the main benefits of and enablers to being involved in the CoP?
7. What are the costs or barriers to being involved in the CoP?
8. Would you recommend joining the CoP to a colleague? Yes/No/Unsure (explore reasons for answer).

(Explore feasibility & acceptability if not picked up on above)

9. What **helped the CoP to work** – what might **energise and interest** you and **keep you or others engaged?**
10. **How confident did you feel that you** were **an active and contributing membe**r of an IPC leads CoP? (more about personal skills, confidence, sharing – not logistics).
11. What **possible risks, challenges or potential pitfalls** can you think of in relation to the CoP?
12. **What will you need to do to enable you to continue to participate regularly in the CoP**? How will a group like this fit into your current workload?

**Specific questions about any IPC practice improvement activity implemented as part of the IMMERSE-2 project:**

13. We’re going to talk in more detail about an example of an IPC practice change activity you led at your workplace.

- Did you use any of the resources from the CoP sessions? *(Prompt: use of the [specify resource discussed in CoP session]? How useful were they?)*
- Who else from your workplace was involved in the practice change activity? Can you describe their role? *Prompt: did you feel supported by your manager? Or other colleagues?*
- How did you identify the practice improvement problem? And determine the knowledge-practice gap?
- Did you assess barriers and facilitators to the practice change? Can you describe this for me?
- How did you select the practice change strategy/ies? Please describe the strategy/ies for me?
- Did you tailor the strategy/ies? Please describe.
- How did you implement the strategy/ies? And monitor their use – how did you measure this? What was/were the outcome(s) of the improvement project?
- What went well? What didn’t go so well?
- If you had your time again, what, if anything, would you do differently?

14. Summary question: What more would you like to share about your IPC role, leading practice improvement or the CoP? (Check if anything else to share about the experiences of practice improvement and/or the CoP).

(Close interview by reiterating key information re summary data, deidentified reporting and how the project will feedback. Provide contact details for IMMERSE-2 team if any further thoughts or questions arise). Thank you for your time today.
